# MiR-29a Suppresses Spermatogenic Cell Apoptosis in Testicular Ischemia-Reperfusion Injury by Targeting TRPV4 Channels

**DOI:** 10.3389/fphys.2017.00966

**Published:** 2017-11-29

**Authors:** Jin-zhuo Ning, Wei Li, Fan Cheng, Wei-min Yu, Ting Rao, Yuan Ruan, Run Yuan, Xiao-bin Zhang, Dong Zhuo, Yang Du, Cheng-cheng Xiao

**Affiliations:** ^1^Department of Urology, Renmin Hospital of Wuhan University, Wuhan, China; ^2^Department of Anesthesiology, Renmin Hospital of Wuhan University, Wuhan, China; ^3^Department of Urology, Wannan Medical College, Wuhu, China

**Keywords:** miR-29a, TRPV4, testicular, IRI, apoptosis

## Abstract

**Background:** MicroRNAs (miRNAs) have emerged as gene expression regulators in the progression of ischemia-reperfusion injury (IRI). Accumulating evidences have indicated miR-29a play roles in myocardial and cerebral IRI. However, the role of miR-29a in testicular IRI has not been elucidated.

**Methods:** Changes in expression of miR-29a and Transient Receptor Potential Vanilloid 4 (TRPV4) in animal samples and GC-1 spermatogenic cells were examined. The effects of miR-29a on spermatogenic cell apoptosis in testicular IRI were analyzed both *in vitro* and *in vivo*.

**Results:** The expression of MiR-29a was negatively correlated with the expression of TRPV4 and significantly downregulated in animal samples and GC-1 cells as testicular IRI progressed. Further studies revealed TRPV4 as a downstream target of miR-29a. Inhibition of miR-29a expression increased the expression of TRPV4 and promoted spermatogenic cell apoptosis, whereas overexpression of miR-29a downregulated TRPV4 expression and suppressed spermatogenic cell apoptosis caused by testicular IRI *in vitro* and *in vivo*.

**Conclusion:** Our results suggest that miR-29a suppresses apoptosis induced by testicular IRI by directly targeting TRPV4.

## Introduction

Testicular torsion is the most common cause of urological emergency in young and adolescent males (Tuglu et al., 2016). Once diagnosed, clinical treatment should be adopted to restore blood flow to the testis within an appropriate time frame (Kim et al., 2016). Testicular torsion/detorsion (T/D) is considered to be the primary pathophysiologic event that induces ischemia-reperfusion injury (IRI), which causes an enhancement of apoptosis and testicular spermatogenesis dysfunction (Meštrović et al., 2014). Therefore, elucidating the mechanism underlying cell apoptosis is critical to helping better understand the progression of testicular IRI and to identification of effective therapeutic targets.

Transient Receptor Potential Vanilloid 4 (TRPV4) is a primary member of the transient receptor potential (TRP) channels family (Fusi et al., 2014). TRPV4 is a non-selective cation channel activated by a wide range of stimuli, including heat, cell swelling, temperature changes, pH, anandamide, arachidonic acid metabolites, and other factors (Vergnolle, 2014). TRPV4 plays a key role in regulating various cellular activities and has been found to be expressed in the kidneys, brain, lungs, skin, heart, liver, and testes (Xu et al., 2009; Wei et al., 2015; Tsuno et al., 2016). Notably, it has been reported that excessive activation of this channel is related to renal, lung, and cerebral IRI (Townsley et al., 2010; Kassmann et al., 2013; Ding et al., 2015), suggesting that TRPV4 may be an important target for mediating reperfusion injury following ischemia in many organs.

MicroRNAs (miRNAs) are endogenous, single-stranded non-coding RNAs with a length of 18–25 nucleotides (Zhang et al., 2012). They are capable of downregulating gene expression by targeting specific mRNAs located mostly in 3′-untranslated-regions (Berezikov et al., 2005), thereby participating in a wide range of biological processes, including cell proliferation, development, differentiation and apoptosis (Zhang et al., 2010; Bao et al., 2012). Previous studies have reported that miRNAs are tightly associated with apoptosis during the progression of IRI. For example, miR-499 alleviates apoptosis by downregulating PDCD4 in myocardial IRI (Zhu et al., 2016), miR-200c regulates apoptosis of hepatic IRI by targeting ZEB1 (Wu et al., 2016), and miR-30 decreases apoptosis in renal IRI by repressing DRP1 (Gu et al., 2016). Although recent studies have shown that miR-29a plays a role in apoptosis during myocardial and cerebral IRI (Ye et al., 2010; Ouyang et al., 2013; Wang et al., 2015), the biological effect of miR-29a in testicular IRI and the interrelation between miR-29a and TRPV4 have not been described.

In the present study, we found that the expression of miR-29a was obviously reduced while TRPV4 expression was significantly enhanced in testicular IRI. Moreover, we identified miR-29a regulates TRPV4 expression by directly binding to it and demonstrated that miR-29a plays a key role in regulating IR-induced apoptosis by targeting TRPV4 in the testes. These results suggest that miR-29a may be used as a potential therapeutic option to inhibit apoptosis during the progression of testicular IRI.

## Materials and methods

### Animals and surgical protocols

All experimental procedures were adhered to the National Institutes of Health Guide for the Care and Use of Laboratory Animals and were approved by the Animal Care and Use Committee of Wuhan University. Male C57BL/6 mice (20–25 g) were obtained from the Hubei Center for Disease Control. Prior to experiments, all rats were caged in a standard temperature-controlled room (22 ± 2°C) and were subjected to alternating 12-h light/dark cycles. They also had free access to food and water. The rats were anesthetized by the intraperitoneal administration of 2% sodium phenobarbital (50 mg kg-1) and were then placed on a homeothermic table to maintain a rectal temperature of 37–38°C. The left testis was twisted 720° clockwise and fixed to the scrotal skin with 5/0 silk. After 1 h of torsion, the testis was allowed to recover to the natural position for 0, 4, 8, 16, or 24 h. In the sham group, the testis was localized via a left-sided scrotal incision. The incision was then sutured with 5/0 silk without additional intervention. Additionally, 1–2 μg of mouse miR-29a agomir and its negative control (RiboBio, Guangzhou, China) was injected into seminiferous tubules using an injection pipette (Liang et al., 2012).

### Oxygen glucose deprivation/reperfusion (OGD/R) in GC-1 cells

Mouse GC-1 spermatogenic cells were purchased from ATCC (American Type Culture Collection, Manassas, VA, USA) and maintained in Dulbecco's modified Eagle's medium**(**DMEM; GIBCO, MA, USA) supplemented with 10% fetal bovine serum (FBS) at 37°C under normoxic conditions (5% CO_2_, 95% O_2_). Cells were transfected with pri-miR-29a, anti-miR-29a, siTRPV4, TRPV4-overexpression(GC-1/TRPV4) and their respective negative controls using Lipofectamine 2000 (Invitrogen, Carlsbad, CA, USA) according to the manufacturer's protocol. Then, 48 h after transfection, cells were maintained under hypoxic conditions with glucose-free DMEM for 3 h. After OGD treatment, GC-1 cells were placed in glucose-containing DMEM under normoxic conditions.

### Plasmid construction and luciferase reporter assays

The putative and mutated miR-29a target binding sequence in TRPV4 were synthesized and cloned into luciferase reporter to generate the wild-type (TRPV4-Wt) or mutated-type (TRPV4-Mut) reporter plasmids. The mutant 3′UTR sequence of TRPV4 was generated using overlap extension PCR, and then both the wild-type and mutant sequences were cloned into a psiCHECK-2 vector (Promega, Madison, WI, USA).

For the luciferase reporter assay, GC-1 cells were seeded on a 24-well plate. The cells were then co-transfected with miR-29a mimics or miR-29a negative control using Lipofectamine 2000 (Invitrogen, USA). Luciferase assay was performed 48 h after transfection using a Dual-Luciferase Reporter Assay System (Promega, Madison, WI, USA).

### Western blot analysis

Proteins from testicular tissues or cultured GC-1 cells were extracted using RIPA Lysis Buffer (P0013B, Beyotime Institute of Biotechnology), and protein concentration was qualified using a bicinchoninic acid assay kit (BCA; Beyotime, Shanghai, China). Briefly, Equivalent amounts of protein samples were separated by 10% sodium dodecyl sulfate-polyacrylamide (SDS-PAGE) gels electrophoresis, and transferred to (polyvinylidene fluoride) PVDF membranes subsequently. After that, the membrane was blocked with 5% non-fat milk in Tris-buffered saline and 0.1% Tween 20 (TBST) buffer and incubated with the following primary antibodies against TRPV4(ab94868; Abcam, Cambridge, UK), caspase-3 (sc7148; Santa Cruz, CA), Bax (sc493; Santa Cruz, CA) and Bcl-2 (sc7382; Santa Cruz, CA) at 4°C overnight. GAPDH was used as an internal control. After being rinsed three times with TBST buffer, the membranes were incubated with secondary antibodies at room temperature for 1 h. Target proteins were visualized using an ECL system kit (Pierce Biotechnology, Beijing, China). Optical densities were detected by enhanced chemiluminescent (ECL) and qualified using ImageJ software (NIH, Bethesda, MD, USA).

### Quantitative real-time PCR

Total RNA was isolated from GC-1 cells and testis samples using TRIzol reagent (Invitrogen Life Technologies, Carlsbad, CA, USA), and the concentration of RNA was determined by a DU800 UV/Vis Spectrophotometer (Beckman Coulter, CA, USA). Total cellular RNAs were reversed transcribed into cDNA using reverse transcription reagent kit (Takara Biotechnology, Dalian China). Real-time quantitative PCR was performed via a Applied Biosystems SYBR Green mix kit and the ABI 7900 Real-Time PCR system (Applied Biosystems Life Technologies, Foster City, CA, USA). Relative miR-29a or TRPV4 mRNA expression were normalized to snRNA U6 (for miRNAs) or GAPDH (for mRNAs), respectively. The quantitative analysis was calculated by using 2^−ΔΔCt^ method (Rao et al., 2013). The primer sequences used are shown in Table 1.

**Table 1:** RT-PCR primer sequences.

**Gene**	**Primer sequences (5′-3′)**
TRPV4	F: CGCTCCTTCCCCGTATTCCT
	R: TTGATGATGCCCAAGTTCTGGTT
miR-29a	F: UAGCACCAUCUGAAAUCGGUUA
	R: ACCGUGCUCGACUUUCCGG
U6 snRNA	F: CTCGCTTCGGCAGCACATATACT
	R: ACGCTTCACGAATTTGCGTGTC
GAPDH	F: ACAGCAACAGGGTGGTGGAC
	R: TTTGAGGGTGCAGCGAACTT

### Immunohistochemistry

The expression of TRPV4, Bax and Bcl-2 was detected by immunohistochemical staining. Tissues were fixed in 4% paraformaldehyde, embedded in paraffin and then cut in 4 μm thickness. Immunohistochemical staining was performed using rabbit polyclonal anti-TRPV4 (ab94868; Abcam, Cambridge, UK), rabbit polyclonal anti-Bax (sc493; Santa Cruz, CA) and mouse monoclonal anti-Bcl-2 (sc7382; Santa Cruz, CA). After being washed three times with PBS, all sections were incubated in diaminobenzidine (DAB) reagents and counterstained with haematoxylin.

### TUNEL assays

A TUNEL assay was performed to evaluate spermatogenic cell apoptosis in testicular tissues using a transferase-mediated dUTP nick-end labeling (TUNEL) method with a detection kit (Roche, Mannheim, Germany) following the manufacturer's protocol. The nuclei that stained brown were considered TUNEL-positive cells. Five visual fields were randomly selected in each slice, and the average number of apoptosis cells per 200 cells was counted. The apoptosis index (AI) was determined as follows: AI = (the number of positive cells/the total number of counted cells) × 100%.

### Cell apoptosis analysis

Cell apoptosis was performed using Annexin V-FITC/Propidium Iodide (PI) staining (BD PharMingen, San Jose, CA, USA). GC-1 cells were seeded in 6-well plates at a concentration of 10^6^ cells mL-1. The cells were labeled with Annexin V-FITC for 5 min in the dark. Then, 5 mg ml-1 PI was added to each sample for 30 min so that flow cytometry could be performed (BD PharMingen, San Jose, CA, USA).

### Statistical analysis

All data are presented as the mean ± SD. Differences were assessed by one–way analysis of variance (ANOVA), followed by all pairwise multiple-comparison procedures using the Bonferroni test. A value of *P* < 0.05 was considered statistically significant. All experiments were performed at least 3 times. Statistical analysis was performed using SPSS 19.0 (SPSS Inc, Chicago, IL, USA).

## Results

### Enhanced expression of TRPV4 in testicular IRI correlates with downregulation of miR-29a expression

**Figure 1:**
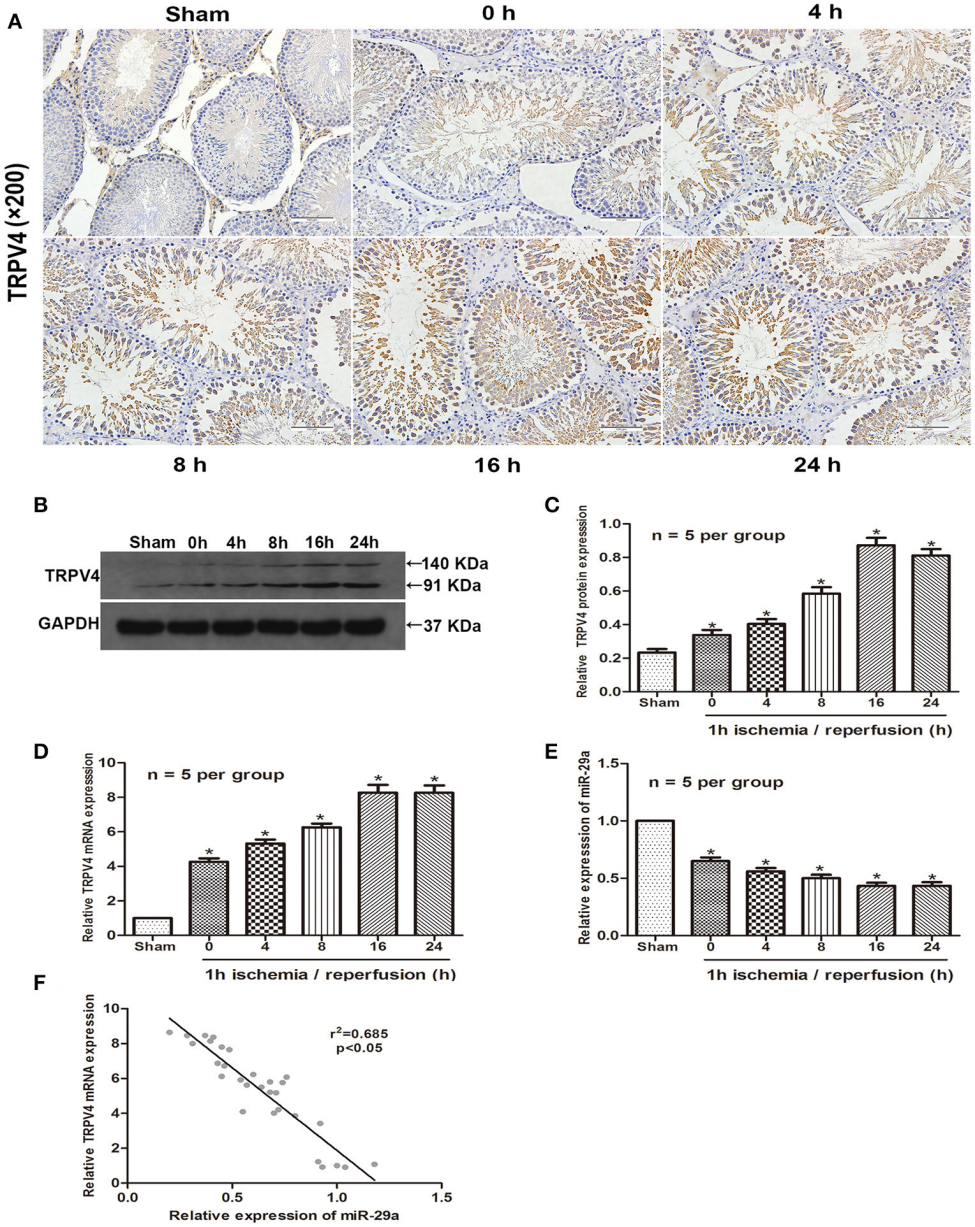
High expression of TRPV4 in testicular IRI correlates with downregulation of miR-29a. **(A)** Immunohistochemistry staining of TRPV4 in the testis exposed to 1 h ischemia followed by reperfusion of different durations. *n* = 5 per group; **(B–E)** QRT-PCR and western blot analysis of miR-29a and TRPV4 expression at different reperfusion times after 1 h ischemia in animal samples. ^*^p < 0.05 vs. sham, *n* = 5 per group; **(F)** A two-tailed Pearson's correlation analysis reveals that the mRNA expression of miR-29a is inversely correlated with the expression of TRPV4 (*p* < 0.05).

To investigate if TRPV4 and miR-29a are involved in testicular IRI, we examined their expression levels in animal samples by immunohistochemistry staining, qRT-PCR and western blot. The samples were collected and assayed for expresssion at 0, 4, 8, 16, or 24 h of reperfusion after 1 h ischemia (Aslan Koşar et al., 2015). The results showed that TRPV4 expression increased gradually and peaked at 16 h of reperfusion compared with the sham group (Figures 1A–D, *n* = 5 per group). On the other hand, miR-29a expression decreased significantly during the progression of IRI (Figure 1E, *n* = 5 per group). A two-tailed Pearson's correlation analysis was performed to further investigate the interrelation between miR-29a and TRPV4 expression (Figure 1F). Therefore, the expression of miR-29a is negatively correlated with the expression of TRPV4 *in vivo*.

### miR-29a is negatively correlated with the expression of TRPV4 *in vitro*

**Figure 2:**
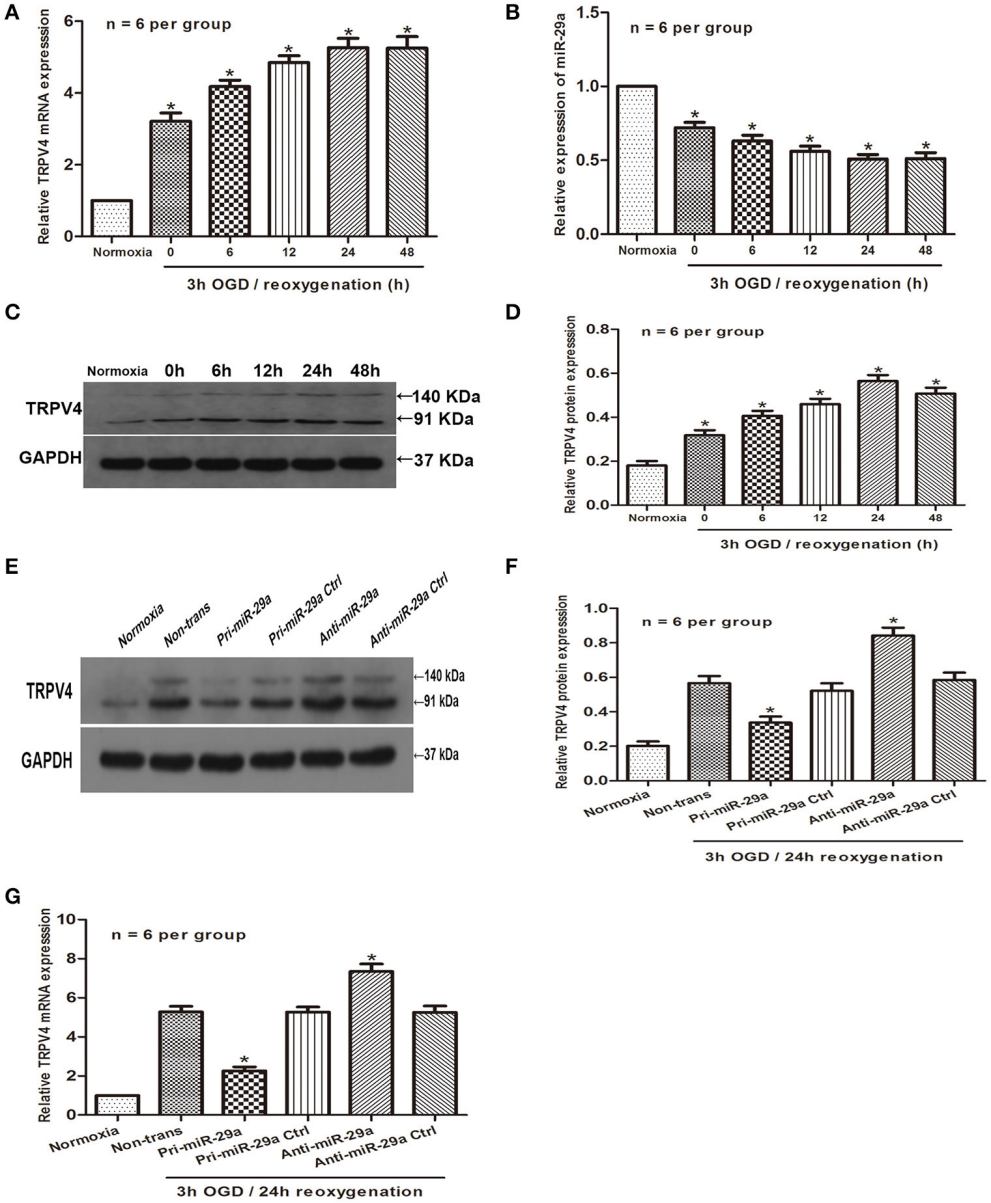
MiR-29a is negatively correlated with the expression of TRPV4 in GC-1 cells. **(A–D)** QRT-PCR and western blot analysis of miR-29a and TRPV4 expression under different reoxygenation conditions after 3 h OGD exposure. ^*^p < 0.05 vs. normoxia, *n* = 6 per group; **(E–G)** GC-1 cells were transfected with pri-miR-29a or anti-miR-29a. Western blot and qRT-PCR analysis were performed to examine TRPV4 mRNA expression in normoxia and 3 h OGD/24 h reoxygenation treatments. ^*^p < 0.05 vs. non-trans (3 h OGD/24 h reoxygenation treatment), *n* = 6 per group.

To further explore the possibility that miR-29a might negatively correlates with the expression of TRPV4 *in vitro*, we examined the expression of TRPV4 and miR-29a in GC-1 cells under different reoxygenation conditions (0, 6, 12, 24, and 48 h) after 3 h OGD exposure. Consistent with the *in vivo* studies, the qRT-PCR and western blot results showed that miR-29a expression was inversely correlated with the expression of TRPV4 at different reoxygenation time intervals (Figures 2A–D, *n* = 6 per group). We next transfected the GC-1 cells with pri-miR-29a and examined TRPV4 expression by western blot and qRT-PCR at 3 h of OGD followed by 24 h of reoxygenation. We found that overexpression of miR-29a led to a significant downregulation of TRPV4 expression. Further, GC-1 cells transfected with a miR-29a inhibitor, displayed a moderate upregulation of TRPV4 expression (Figures 2E–G, *n* = 6 per group).

### Influence of miR-29a and TRPV4 on GC-1 cell apoptosis *in vitro*

**Figure 3:**
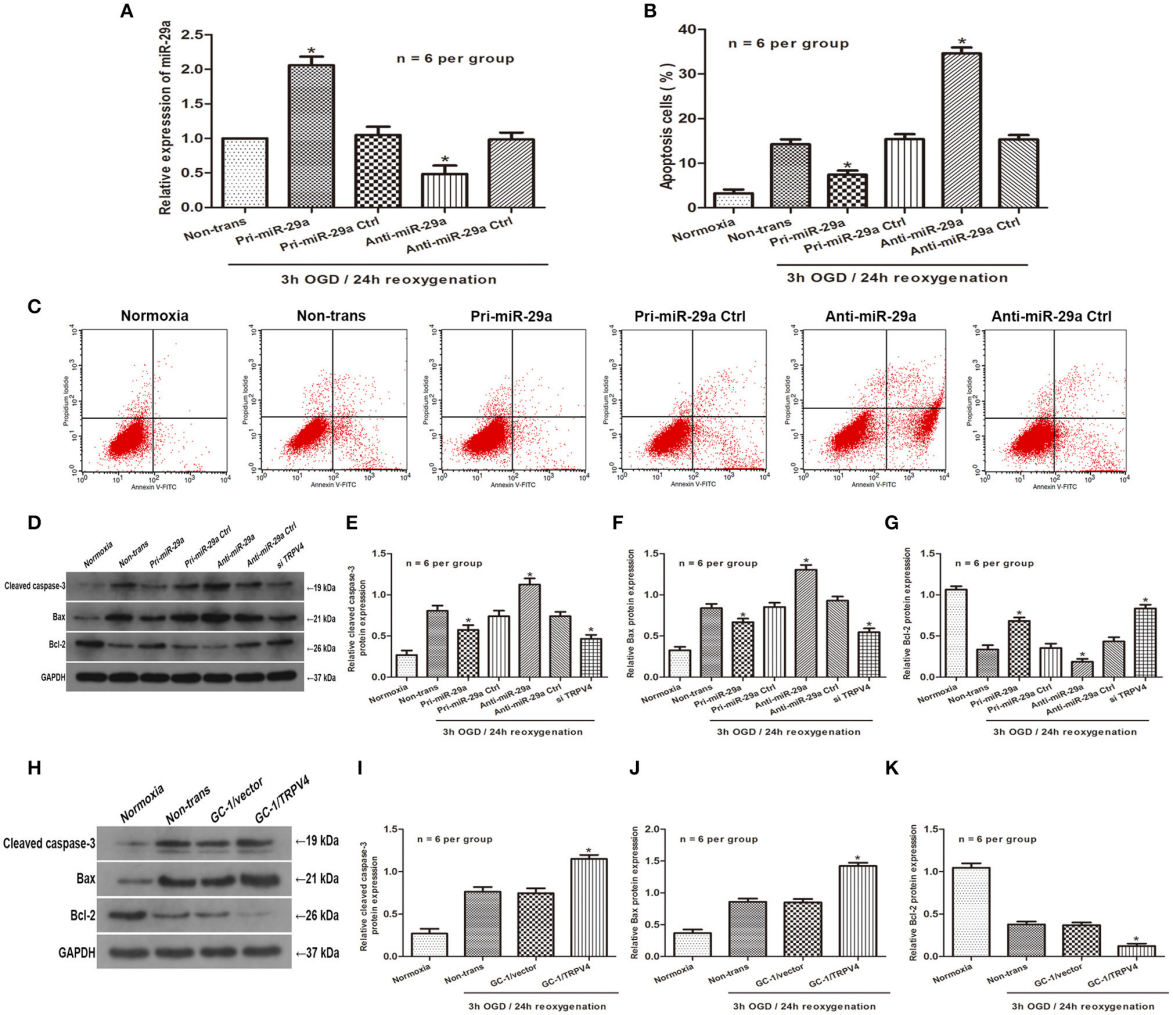
Influence of MiR-29a and TRPV4 on GC-1 cell apoptosis *in vitro* **(A)** qRT-PCR assays were performed to analyze the expression level of miR-29a after transfection with pri-miR-29a or anti-miR-29a in 3 h OGD/24 h reoxygenation treatments. ^*^p < 0.05 vs. non-trans, *n* = 6 per group; **(B,C)** Flow cytometry assays were performed to show the cell apoptosis after transfection with pri-miR-29a or anti-miR-29a in normoxia and 3 h OGD/24 h reoxygenation treatments. ^*^p < 0.05 vs. non-trans, *n* = 6 per group; **(D–K)** Cleaved caspase-3, Bax and Bcl-2 protein levels transfected with pri-miR-29a, anti-miR-29a, TRPV4 siRNAs, or TRPV4-overexpression(GC-1/TRPV4) were examined in normoxia and 3 h OGD/24 h reoxygenation treatments by western blot analysis. ^*^p < 0.05 vs. non-trans, *n* = 6 per group.

To investigate the effect of miR-29a on OGD/R cell injury, we used pri-miR-29a and anti-miR-29a to change miR-29a levels in GC-1 cells. Indeed, pri-miR-29a and anti-miR-29a markedly increased and decreased miR-29a levels at 3 h of OGD/24 h of reoxygenation, respectively when compared with their negative controls (Figure 3A, *n* = 6 per group). Flow cytometry data also showed that GC-1 cell apoptosis was induced by 3 h of OGD/24 h of reoxygenation. Transfection of pri-miR-29a inhibited cell apoptosis, while transfection of anti-miR-29a promoted GC-1 cell apoptosis induced by 3 h of OGD/24 h of reoxygenation (Figures 3B,C, *n* = 6 per group). In addition, western blot analysis showed that overexpression of miR-29a and knockdown of TRPV4 decreased the expression of Bax and caspase-3 and increased the expression of Bcl-2, respectively. Consistent with this result, inhibition of miR-29a and overexpression of TRPV4 in GC-1 cells resulted in an increase in Bax and caspase-3 levels and a decrease in Bcl-2 expression, respectively (Figures 3D–K, *n* = 6 per group). These results suggest that miR-29a suppresses cell apoptosis and TRPV4 promotes cell apoptosis *in vitro*.

### miR-29a directly targets TRPV4 and alleviates apoptosis *in vitro*

**Figure 4:**
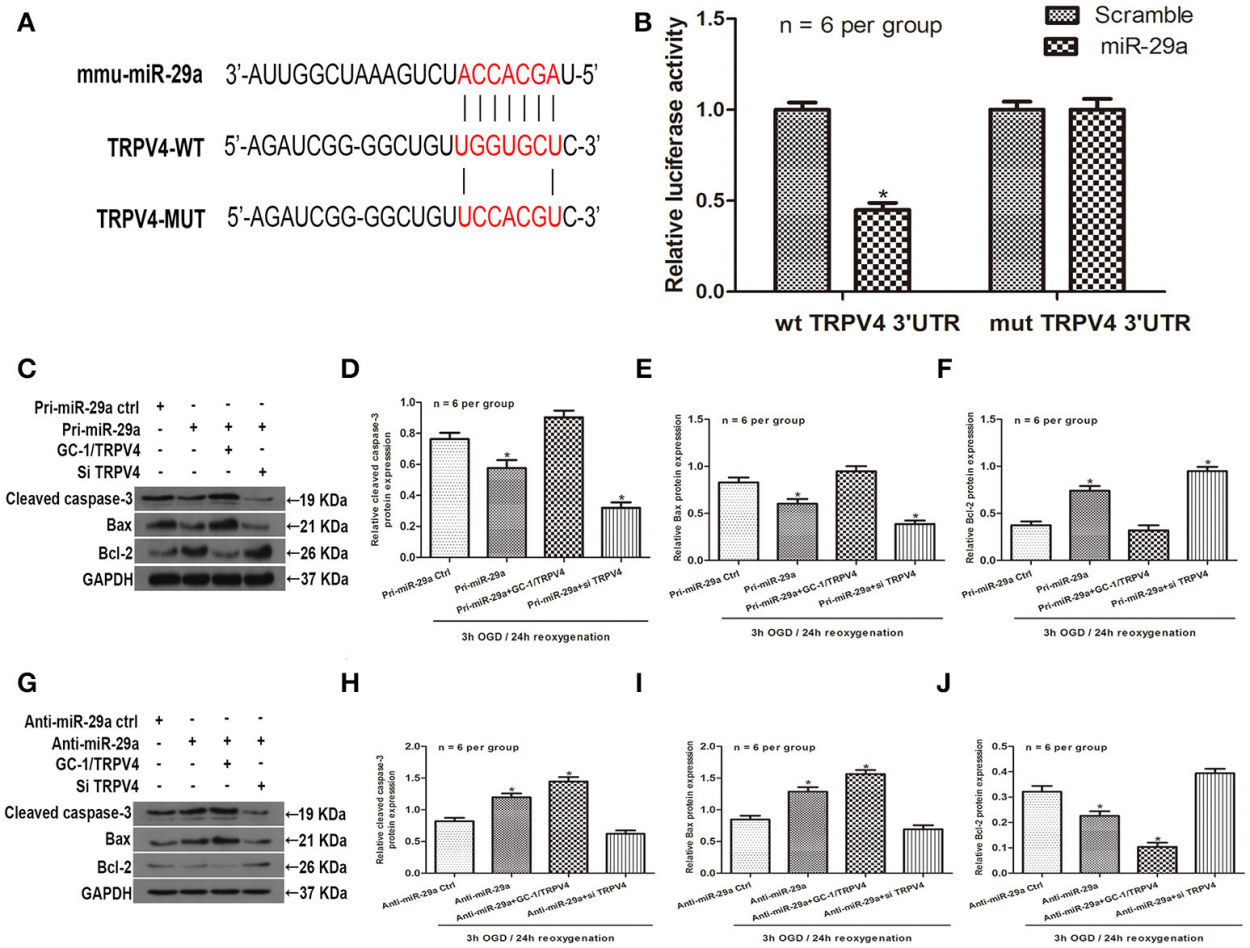
MiR-29a directly targets TRPV4 and alleviates apoptosis *in vitro*. **(A)** Sequence alignment of predicted miR-29a binding sites within the TRPV4 3′UTR and its mutated sequence for luciferase reporter assay; **(B)** Luciferase reporter assay was performed in GC-1 cells that were co-transfected with pri-miR-29a and reporter vectors containing TRPV4 3′UTR or mutated TRPV4 3′UTR. Relative luciferase activities are presented. ^*^p < 0.05, *n* = 6 per group. **(C–F)** Cleaved caspase-3, Bax and Bcl-2 protein levels of GC-1 cells co-transfected with pri-miR-29a and TRPV4 siRNAs or TRPV4 overexpression (GC-1/TRPV4) in 3 h OGD/24 h reoxygenation treatments by western blot analysis. ^*^p < 0.05 vs. pri-miR-29a ctrl, *n* = 6 per group. **(G–J)** Cleaved caspase-3, Bax and Bcl-2 protein levels of GC-1 cells co-transfected with anti-miR-29a and TRPV4 siRNAs or TRPV4-overexpression(GC-1/TRPV4) in 3 h OGD/24 h reoxygenation treatments by western blot analysis. ^*^p < 0.05 vs. anti-miR-29a ctrl, *n* = 6 per group.

To further explore the relationship between miR-29a and TRPV4, an *in silico* prediction was performed using open access software (TargetScan, PicTarget, and miRanda). A putative binding site for miR-29a was identified within the 3′UTR of TRPV4. To verify this prediction, we cloned a luciferase reporter sequence in the 3′UTR of TRPV4, which contains the putative miR-29a binding sites. A mutant reporter vector of the 3′UTR of TRPV4 containing luciferase reporter was used as negative control. Data from luciferase reporter assay showed that overexpression of miR-29a significantly decreased reporter vector activity of TRPV4 3′UTR in GC-1 cells but had no effect on the mutated reporter vector (Figures 4A,B, *n* = 6 per group). To further investigate whether miR-29a regulates apoptosis in testicular IRI via targeting TRPV4, we employed immunoblotting assays to determine apoptosis related proteins after cells were transfected with pri-miR-29a or anti-miR-29a and TRPV4 siRNAs or TRPV4 overexpression (GC-1/TRPV4). We found that overexpression of miR-29a markedly suppresses apoptosis, whereas TRPV4 overexpression abrogated such decrease in apoptosis induced by miR-29a (Figures 4C–F, *n* = 6 per group). On the other hand, knockdown of miR-29a led to an increase in apoptosis, while which could be rescued by TRPV4 inhibition (Figures 4G–J, *n* = 6 per group). Together, these results suggest that miR-29a directly targets TRPV4 and alleviates apoptosis *in vitro*.

### miR-29a inhibits spermatogenic cell apoptosis via downregulation of TRPV4 *in vivo*

**Figure 5:**
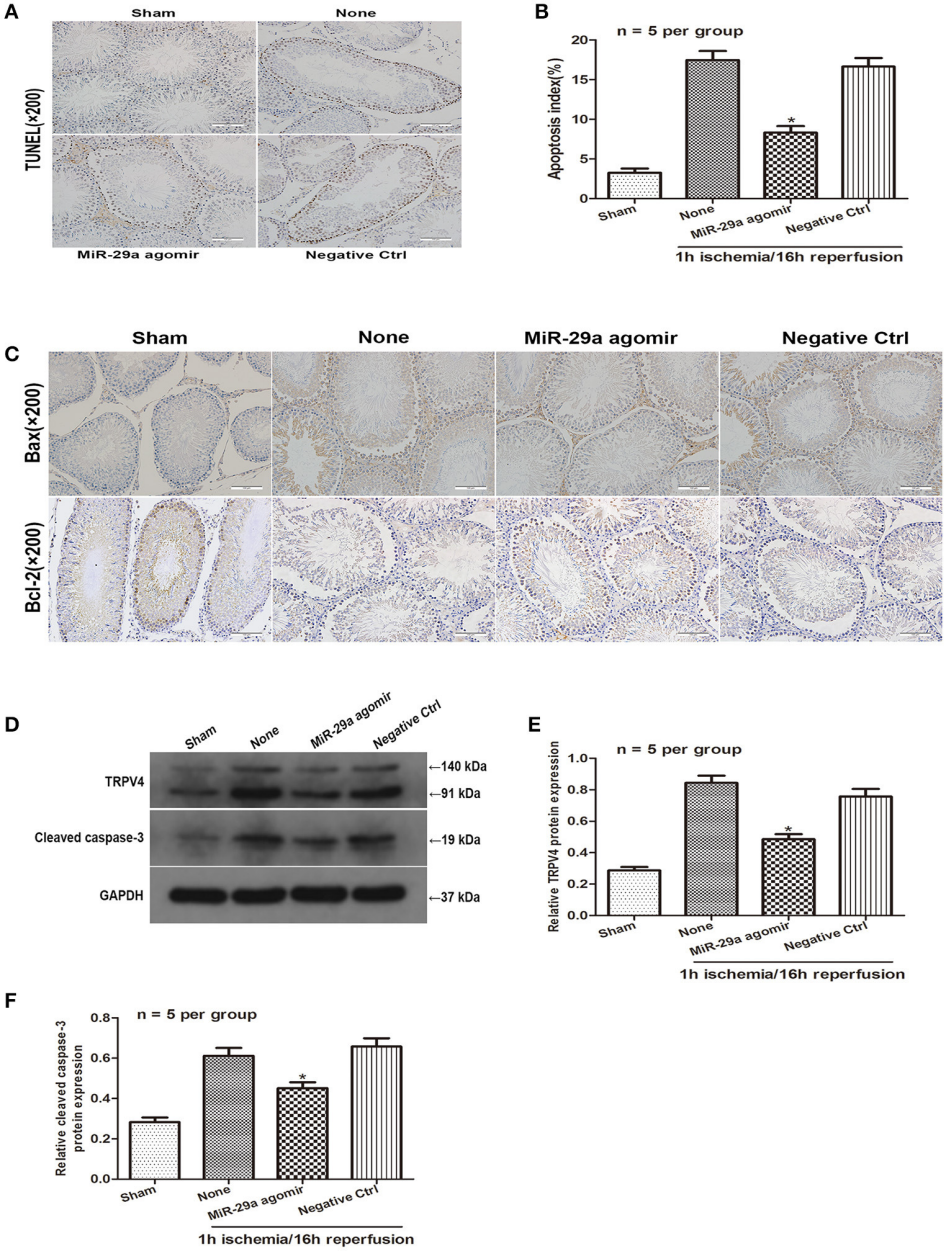
MiR-29a inhibits apoptosis of testicular IRI via downregulation of TRPV4 *in vivo*. **(A,B)** TUNEL assays were performed to investigate the cell apoptosis after injection with miR-29a agomir and its negative ctrl at 16 h of reperfusion after 1 h ischemia. ^*^p < 0.05 vs. none(1 h ischemia/16 h reperfusion treatment), *n* = 5 per group; **(C)** Immunohistochemistry staining of Bax and Bcl-2 after injection with miR-29a agomir and its negative ctrl at 1 h of ischemia/16 h of reperfusion. *n* = 5 per group; **(D–F)** TRPV4 and Cleaved caspase-3 protein level expression after injection with miR-29a agomir and its negative ctrl were detected at 1 h of ischemia followed by 16 h of reperfusion by western blot analysis. ^*^p < 0.05 vs. none, *n* = 5 per group.

To further determine the effect of miR-29a expression on spermatogenic cell apoptosis *in vivo*, miR-29a agomir was injected into the seminiferous tubules to increase miR-29a expression. A TUNEL assay was used to determine whether miR-29a could affect spermatogenic cell apoptosis in response to a 1 h of ischemia/16 h of reperfusion treatment. The TUNEL assay showed no obvious apoptotic cells in the sham group. Compared with negative control group, the effect of miR-29a overexpression dramatically reduced the number of apoptotic cells (Figures 5A,**B**, *n* = 5 per group). Immunohistochemistry staining revealed that overexpression of miR-29a resulted in lower expression levels of Bax and a higher expression level of Bcl-2 in comparison with negative control group (Figure 5C, *n* = 5 per group). Moreover, western blot data showed that overexpression of miR-29a decreased protein levels of TRPV4 and caspase-3 when compared to the negative control group (Figures 5D–F, *n* = 5 per group). Together, our results suggest that miR-29a suppresses apoptosis *in vivo*.

## Discussion

The pathophysiological mechanisms of testicular T/D result from direct damage induced by ischemia during torsion and a secondary effect attributed to enhanced blood flow with reperfusion during detorsion (Huang et al., 2012). Testicular IRI triggers apoptosis-related signaling pathways and further negatively affects spermatogenesis, eventually leading to male infertility (Hadziselimovic et al., 1998; Meštrović et al., 2014). The two major factors affecting testicular damage are the degree and duration of spermatic cord torsion (Cvetkovic et al., 2015). Timely treatment of testis torsion is a crucial issue, and currently both manipulative reduction and orchiectomy treatment are known to cause injury to the bilateral testis (Dursun et al., 2015). Thus, understanding the mechanisms underlying apoptosis in testicular IRI and exploring new molecular interactive targets will help to develop effective therapeutics. Previous studies have demonstrated that miRNA can regulate apoptosis process in organ IRI by targeting mRNAs of specific genes (Berezikov et al., 2005). Based on this information, we tried to identify miRNAs that bind the TRPV4 gene and to determine the regulatory relationship in the progression of testicular IRI.

MicroRNA expression is highly related to apoptosis in the progression of organs IRI. It has been shown that abnormally expressed miRNAs can regulate RNA networks during IRI progression, and a single miRNA may function in pro-apoptosis or anti-apoptosis roles under different contexts (Andreeva et al., 2015). Previous studies have demonstrated that overexpression of miR-29a promotes myocardial apoptosis by directly targeting IGF-1 and Mcl-1 depending on activation of P13K/Akt signaling pathways (Ye et al., 2010; Wang et al., 2015). Furthermore, a recent study suggested that miR-29a suppresses apoptosis by negatively regulating PUMA in cerebral IRI (Ouyang et al., 2013). In our studies, we analysed animal samples at different time points of reperfusion. Our data demonstrated that miR-29a expression is significantly downregulated in the progression of testicular IRI and that lower expression of miR-29a is negatively correlated with expression of TRPV4 channels.

TRPV4 has been reported to be upregulated during the progression of IRI and to be involved in apoptosis via multiple signaling pathways (Ye et al., 2012; Hong et al., 2016). For example, TRPV4 expression is upregulated during the progression of IRI and involved in apoptosis via multiple signaling pathways (Jie et al., 2015). Inhibition of TRPV4 reduces neurological injury after cerebral infarction, and leads to apoptosis of mouse retinal ganglion cells, which may contribute to the activation of Ca2^+^-dependent signaling pathways (Ryskamp et al., 2011). In our *in vitro* studies, we further confirmed the opposite relationship between TRPV4 and miR-29a expression in the GC-1 cells. We found that overexpression of miR-29a suppressed TRPV4 expression and that inhibition of miR-29a promoted TRPV4 expression. More important, we have identified TRPV4 as a direct downstream target of miR-29a using a luciferase reporter assay. These data provide evidence that miR-29a negatively regulates the expression of TRPV4 in the GC-1 cells, which is consistent with findings from *in vivo* studies.

Apoptosis is a form of cell death based on genetic mechanisms and plays an important role by inducing a series of pathophysiologic changes (Vaux and Korsmeyer, 1999), and it has been generally accepted that the mitochondrial signaling pathway is the major channel for apoptosis, which is regulated by precise gene expression (Desagher and Martinou, 2000). The Bcl-2 family is composed of pro-apoptotic factors (e.g., Bax) and anti-apoptotic factors (e.g., Bcl-2). The ratio of Bcl-2/Bax is a regulator of spermatogenic cell apoptosis and determines the extent of apoptosis in spermatogenic cells exposed to damage (Liang et al., 2013). Moreover, caspase-3 is an inactive zymogen located in the cytoplasm and serves as the convergence point of multiple apoptotic stimuli signals. Its activation signals irreversible commitment to cell apoptosis, leading to changes in cell shrinkage, chromatin condensation and DNA fragmentation (Zheng et al., 2008). In our *in vitro* studies, we have demonstrated that overexpression of miR-29a and knockdown of TRPV4 reduce cell apoptosis, while inhibition of miR-29a and overexpression of TRPV4 have an opposite effect. Moreover, data from *in vivo* studies also support that increased expression of miR-29a produces a decrease in apoptosis by downregulating the TRPV4 gene expression. Together, these data suggest that miR-29a-mediated inhibition of TRPV4 is associated with cell apoptosis in testicular IRI.

## Conclusions

In conclusion, our study revealed a negative correlation between the expression of miR-29a and TRPV4 during the progression of IRI. In addition, we showed that miR-29a alleviated apoptosis by directly targeting TRPV4 both *in vitro* and *in vivo*. These findings suggest that miR-29a might serve as an effective therapeutic target to suppress spermatogenic cell apoptosis in testicular IRI.

## Author contributions

JN and WL, Conception and design of the study, data collection and analysis, manuscript writing. FC, WY, TR, and DZ, Design of the study, critical revision, supervised all phases of the study. RY, YR, XZ, YD, and CX, Data collection.

### Conflict of interest statement

The authors declare that the research was conducted in the absence of any commercial or financial relationships that could be construed as a potential conflict of interest. The reviewer RDM and handling Editor declared their shared affiliation.
